# The Principle of Alternate Possibilities as Sufficient but not Necessary for Moral Responsibility: A way to Avoid the Frankfurt Counter-Example

**DOI:** 10.1007/s11406-016-9690-2

**Published:** 2016-02-27

**Authors:** Garry Young

**Affiliations:** Division of Psychology, Nottingham Trent University, Burton Street, Nottingham, NG1 4BU UK

**Keywords:** Frankfurt counter-example, Twin world condition, Alternate possibilities, Moral responsibility

## Abstract

The aim of this paper is to present a version of the principle of alternate possibilities (PAP) which is not susceptible to the Frankfurt-style counter-example. I argue that PAP does not need to be endorsed as a necessary condition for moral responsibility and, in fact, presenting PAP as a sufficient condition maintains its usefulness as a maxim for moral accountability whilst avoiding Frankfurt-style counter-examples. In addition, I provide a further sufficient condition for moral responsibility – the twin world condition – and argue that this provides a means of justifying why the protagonist in Frankfurt-style scenarios (e.g., Jones) is still felt to be morally responsible. I conclude with the claim that neither the amended PAP nor the twin world condition is necessary for the ascription of moral responsibility; rather, what is necessary is simply that one of these conditions is satisfied.

## Introduction

Harry Frankfurt describes the principle of alternate possibilities (PAP) as follows: “A person is morally responsible for what he has done *only if* he could have done otherwise” (Frankfurt 1969, p.829; emphasis added). Here, Frankfurt presents the act of ‘being able to do otherwise’ as a necessary condition for the ascription of moral responsibility. He then sets out, using his now famous counter-example involving Jones and Black, to show that PAP is false. The legitimacy of Frankfurt’s challenge to PAP – as necessary for the ascription of moral responsibility – has been debated extensively over the years. A number of theorists have concerned themselves with the question of whether alternate possibilities nevertheless remain – amounting to a flicker of freedom (Fischer 2002) – and therefore whether Frankfurt-style counter-examples in general constitute a legitimate challenge to PAP (see Fischer 1994; Janzen 2013; Leon and Tognazzini 2010; Palmer 2011; Schnall 2001; Young 2007, by way of a small selection of papers on this). Despite the intricacies of some of these continuing debates, focus remains on PAP as a *necessary* condition for moral responsibility.

The aims of this paper is to argue that, as a maxim for moral responsibility, endorsing PAP as a sufficient condition is as effective as presenting it as a necessary condition. Moreover, PAP as a sufficient condition – hereafter PAP_(s)_ – has the advantage of being able to withstand Frankfurt-style counter-examples without, I contend, losing its efficacy as a maxim for moral responsibility. In other words, there is nothing to be lost in terms of our commitment to understanding the conditions for moral accountability by treating PAP as a sufficient condition, but much to be gained: for not only is PAP_(s)_ impervious to Frankfurt-style counter-examples (as noted) but, perhaps more importantly, it opens up debate on what other factors are likewise sufficient for moral responsibility – as the Frankfurt-style scenarios seem to indicate – and which, if any, is necessary.

In the next section I present the case for PAP_(s)_ as a maxim for moral accountability; arguing that it is just as informative to the task of ascribing moral responsibility as PAP. I follow this with a discussion on how PAP_(s)_ is able to withstand the challenge set by Frankfurt-style scenarios, before moving on to consider a further sufficient condition for moral responsibility. This further condition – which I am calling ‘twin world’ – explains why we are still justified in assigning moral responsibility to an agent who fails to satisfy PAP in the manner described within Frankfurt’s counter-example. I conclude by arguing that while neither PAP_(s)_ nor the twin world condition is necessary for an ascription of moral responsibility, given that each nevertheless makes reference to alternate possibilities in some form or other (as yet to be discussed), what is necessary is that *one* of these condition is satisfied: for where both fail to be satisfied, one cannot hold the agent of action morally responsible. What this means is that alternate possibilities are necessary in *some form* for the ascription of moral responsibility but not necessarily in the form by which one could have acted otherwise on a given occasion, as the original formulation of PAP demands.

## PAP as Sufficient for Moral Responsibility

As a sufficient condition for moral responsibility, the principle of alternate possibilities reads as follows:

### PAP_(s)_

A person is morally responsible for what they have done if they could have done otherwise.

In stating that S *could* have done otherwise, I am endorsing the view (much as I take Frankfurt to have done when formulating PAP) that:

- It is possible for S to do otherwise insofar as S is both physically and mentally capable of doing otherwise (including being aware of alternate possibilities that he/she (hereafter ‘he’) is able to select/perform).
- All external conditions required to enable alternate possibilities are satisfied.

Where S engages in action E, if (a) and (b) are satisfied then S is morally responsible for E. Stating this does not negate the possibility that S could still be held morally responsible for E, however, even where (a) and/or (b) fail to be satisfied, should another sufficient condition (as yet unspecified) be met. By making PAP sufficient rather than necessary for moral responsibility, it is still the case that anyone who satisfies (a) and (b) is held to account, morally. The advantage of PAP_(s)_ over Frankfurt’s original PAP, however, is that should (a) and/or (b) fail to be satisfied then this does not rule out the possibility that S is nevertheless morally responsible for E. Under the terms of the original PAP, the possibility just described would not exist; which of course Frankfurt, through the use of his counter-example, tried to show was an erroneous outcome: for, intuitively, we feel that S is still responsible for what he does, even though he could not have done otherwise.

In short, where the conditions inherent within both PAP_(s)_ and Frankfurt’s original PAP are met – namely, clauses (a) and (b) – then PAP_(s)_, as a maxim for moral accountability, is as effective as PAP at ascribing moral responsibility. PAP_(s)_ is, however, not vulnerable to Frankfurt’s counter-example, as I shall now demonstrate.

## Fending off the Frankfurt-Style Counter-Example

For those unfamiliar with Frankfurt’s original and much cited counter-example to PAP, it reads as follows:

Suppose someone – Black, let us say – wants Jones to perform a certain action. Black is prepared to go to considerable lengths to get his way, but he prefers to avoid showing his hand unnecessarily. So he waits until Jones is about to make up his mind what to do, and does nothing unless it is clear to him (Black is an excellent judge of such things) that Jones is going to decide to do something other than what he wants him to do. If it does become clear that Jones is going to decide to do something else, Black takes effective steps to ensure that Jones decides to do, and that he does do, what he wants him to do. Whatever Jones’s initial preferences and inclinations, then, Black will have his way. (Frankfurt 1969, p.835)^1^

In the scenario above, involving Jones and the counterfactual intervener, Black – or even in similar Frankfurt-style scenarios – arguably clauses (a) and (b) are violated. Jones is incapable of making a decision that differs from what Black wants him to decide, and subsequently is incapable of doing other than what Black wants him to do. Thus, Jones cannot satisfy (a). One might even say that the presence of a counterfactual intervener means that the environment does not enable alternate possibilities; so (b) is not satisfied. As is well documented, this scenario led Frankfurt to conclude that PAP is false because, intuitively, we want to assign moral responsibility to Jones in this situation even though clauses (a) and/or (b) are not met and so he could not have done otherwise: a necessary condition for moral responsibility under the original PAP.

PAP_(s)_ is not vulnerable to Frankfurt’s or similar counter-examples. We can allow that clauses (a) and (b) are violated because such violation does not negate the possibility that Jones is nevertheless morally responsible for his action: for some other condition (as yet to be identified) could exist that is sufficient to justify the claim that Jones is morally responsible, even though he could not have done other than he did.

## What Other Condition is Sufficient for the Ascription of Moral Responsibility?

Typically, we want to ascribe moral responsibility to Jones because *he* decided to carry out an act that, let us say, we judge to be morally reprehensible. He decided to do this without the intervention of Black, and so made this decision oblivious to the fact that he could not have decided nor done other than he did. We have to be careful, of course, about how such a description is formally presented. We cannot simply say that S is morally responsible because *he* decided to do E and then did E (hereafter, doing E includes deciding to do E). Where S is coerced into doing E, for example, we would not want to say that S is morally responsible, especially where the coercion is such that it eliminates all morally acceptable alternatives. An example of this would be a form of ‘Sophie’s choice’ whereby S is ordered at gun point to kill one of his two children. If he refuses then both will be killed. In this case, there are alternate possibilities, of course, thereby making it possible for S to do other than he did; but there are no alternate possibilities that do not involve a morally repugnant outcome; meaning that S cannot engage in any action that does not result in an immoral act occurring. When discussing alternate possibilities, then, the notion of ‘alternate possibilities’ should be understood to mean that there is at least one alternate possibility that is considered morally praiseworthy, or at the very least not immoral.

Given that Jones (in a typical Frankfurt-style scenario) has no alternate possibilities available to him, and therefore could not have done other than he did, how might one formulate the condition under which we would be justified in attributing moral responsibility to him? By way of a response, consider the following:

### The Twin World Condition

Where S’s action E in world W_1_ (a world without alternate possibilities, owing to the possibility of intervention) and S’s action E in twin world W_2_ (which differs from W_1_ only insofar as it is a world with alternate possibilities) are congruent then S in either world is morally responsible for E.

To illustrate how one might apply the twin world condition, consider scenarios (1) and (2):

1. ᅟ

- W_1_ (No alternate possibilities): S does E (an immoral act)
- W_2_ (alternate possibilities are available): S does E (an immoral act)

(2) ᅟ

- W_1_ (No alternate possibilities): S does E (a moral act)
- W_2_ (alternate possibilities are available): S does E (a moral act)

Whatever E happens to be, it is congruent across the different worlds within each respective scenario: E is congruent across W_1_ and W_2_ within scenario 1 and congruent across W_1_ and W_2_ within scenario 2. But E differs across the scenarios themselves: for it just so happens that S engages in an immoral act in (1) but a moral act in (2). In both (1) and (2), S is morally responsible for E in W_2_ because, within each scenario, S satisfies PAP_(s)_ in W_2_; and, as we have seen, PAP_(s)_ provides sufficient justification for the ascription of moral responsibility. But in each scenario, S is also morally responsible for E in W_1_ in virtue of the fact that S’s action in W_1_ is *congruent* with S’s action in W_2_ (it is congruent with what S would have done in a world with alternate possibilities). Irrespective of which world one is referring to, then, S should be morally condemned for performing E in (1) but morally praised for the action carried out in (2).

In the context of Frankfurt’s scenario, which I am equating to W_1_, it is made clear that Jones did what he did irrespective of Black’s wishes, and therefore without the need for Black’s intervention. As Jones did what he would have done in the absence of Black, he acted in a manner congruent with what he would have done in a world with alternate possibilities (which is equivalent to W_2_). Mapping my twin world condition onto the Frankfurt scenario, we should understand Jones’s action E to be congruent across W_1_ and W_2_. Jones is therefore morally responsible for his action (even in a world corresponding to W_1_) in virtue of doing what he would have done in a world where alternate possibilities exist (namely, W_2_). PAP_(s)_ and the twin world condition are therefore both sufficient to ascribe moral responsibility to Jones or S or whoever, but PAP_(s)_ is not necessary because moral responsibility could still be attributed to the subject where PAP_(s)_ fails to be satisfied but (and therefore because) the twin world condition is satisfied.

Now, while PAP_(s)_ is not necessary for the attribution of moral responsibility, it could be argued that the necessity of alternate possibilities nevertheless remains, as evidenced by the twin world condition. To explain: despite being unable to do otherwise (and so failing to satisfy PAP_(s)_), if Jones (or S or whoever) is still said to be morally responsibly in virtue of his action’s congruence with what his action *would have been* in a world with alternate possibilities (namely, W_2_) then this means that we are still reliant on alternate possibilities in some form to justify our moral pronouncements. It seems that we have not escaped the need for alternate possibilities, thereby making alternate possibilities necessary. If this is the case, then we are in a position that is ultimately no different to the original formulation of PAP in which it is necessary that the subject could have done otherwise.

There is, however, a subtle but important distinction between PAP in its original formulation – as a necessary condition – and the manner in which the notion of alternate possibilities is presented within the twin world condition. For the ascription of moral responsibility, it is my contention that it is not necessary that S *could have done otherwise*, as required by PAP in its original formulation. Certainly, the ‘could have done otherwise’ clause is indicative of *one role* for alternate possibilities (perhaps the most obvious): namely, making available other options at a given time. But this is not a necessary one; although, where satisfied, it is sufficient for the ascription of moral responsibility, as I have discussed. It is not necessary because, where alternate possibilities are not available (as in, a Frankfurt-style scenario akin to W_1_), and therefore S could not have done otherwise on a given occasion, one can still ascribe moral responsibility through an enforcement of the twin world condition. Yet, in the twin world condition, the role played by alternate possibilities is not one of ‘being available to S′, such that he could have done other than he did on a particular occasion (as required by PAP_(s)_ and W_2_), because, of course, they are not available. Instead, the role played by alternative possibilities in the twin world condition is more *subjunctive*, insofar as what is described is a possibility rather than an actuality. In this sense, it is more akin to the clause ‘if it were the case’ rather than ‘given that it is’.

In the twin world condition, S is ascribed moral responsibility because alternate possibilities, *should they have been available*, would not have changed S’s decision making. This is something that one can assess through the twin world condition in the absence of the current availability of alternative possibilities, and therefore even when it is not possible for S to have done other than he did on a given occasion. I therefore seek to distinguish between alternative possibilities in the form of the alleged necessity of being able to do otherwise on a given occasion, which S cannot satisfy in Frankfurt-style scenarios, and which I do not consider to be necessary for the ascription of moral responsibility (although, to reiterate, I accept it is sufficient), and the role played by alternative possibilities in the twin world condition. In the latter case, alternative possibilities are used in a more hypothetical context to assess if S would have been affected by their availability to the point of selecting one of these options *should they have been available*. Whether this would have been the case is evidenced by the congruence (or lack thereof) between E in W_1_ and W_2_.

For the ascription of moral responsibility, what is necessary is that alternate possibilities have some role to play in whatever conditions we devise to determine who is responsible and who is not, and why. The manner in which alternate possibilities are presented differs, though, within each of the sufficient conditions discussed. Putting all of this together, we can now see how each sufficient condition contains a different form of, and therefore a different role for, alternate possibilities. Recall:

- PAP_(s)_: A person is morally responsible for what they have done if they could have done otherwise.

And:

- **The twin world condition:** Where S’s action E in world W_1_ (a world without alternate possibilities, owing to the possibility of intervention) and S’s action E in twin world W_2_ (which differs from W_1_ only insofar as it is a world with alternate possibilities) are congruent then S in either world is morally responsible for E.

PAP_(s)_ is not necessary for the ascription of moral responsibility because moral responsibility could still be ascribed to S even where S could not have done otherwise, as the twin world condition illustrates. The twin world condition is likewise not necessary because one may not be required to compare S’s action in W_1_ with W_2_, owing to the fact that S is not performing E in a world without alternate possibilities (e.g., W_2_); in which case, PAP_(s)_ applies.

Inherent within each of the aforementioned sufficient conditions is *a* role for alternate possibilities. Alternate possibilities (or at least an alternate possibility) are therefore necessary in some form for the ascription of moral responsibility (as we have seen), but acknowledging this is not the same as saying that it is necessary that S could have done otherwise on a given occasion (as is required by the original PAP). As a role for alternate possibilities is necessary in some form for the ascription of moral responsibility, and as different forms of this role are inherent within the two sufficient conditions described above, what is necessary for the ascription of moral responsibility is that one of these aforementioned sufficient conditions is satisfied. Where S satisfies PAP_(s)_, S is morally responsible for E; where this is not the case then S should still be ascribed moral responsibility if the twin world condition is satisfied. Where neither is satisfied then S is not morally responsible for performing E.

## The Case of Incongruence and Dispensing with the Flicker of Freedom

Frankfurt-style scenarios illustrate the condition under which PAP_(s)_ is not satisfied. What would have to occur in order for the twin world condition not to be satisfied? To illustrate:

(3) ᅟ

- W_1_ (No alternate possibilities): S does E (an immoral act)
- W_2_ (alternate possibilities are available): S does E (a moral act)

In twin world scenarios, all must be equal apart from the one thing that distinguishes each world from the other which, in this case, is the presence or absence of alternate possibilities. This means that, ceteris paribus, what S would have done in W_2_ is what S would do in W_1_ in the absence of intervention, otherwise the coherence of the twin world scenario falls apart. Where this is not the cases, as expressed within (3) then we must conclude that intervention has occurred in W_1_, thereby explaining the incongruence across worlds. As such, S fails to satisfy both PAP_(s)_ and the twin world condition, and should not therefore be held to account, morally. Likewise:

(4) ᅟ

- W_1_ (No alternate possibilities): S does E (a moral act)
- W_2_ (alternate possibilities are available): S does E (an immoral act)

Here, S should not be morally praised for E in W_1_ as we must conclude that some form of intervention has occurred thereby making S’s action in W_1_ incongruent with what he would have done in W_2_. Again, S fails to satisfy PAP_(s)_ and the twin world condition, and so is not morally responsible for E, even though E is a good thing to have done in this case.

Finally, the twin world condition allows another aspect of the debate over the Frankfurt-style counter-examples to be side-stepped: namely, whether the ‘sign’ used as an indicator of an impending decision – which the counterfactual interveners uses as a marker of whether to intervene or not – is sufficiently robust to count as evidence of an alternate possibility (Elzein 2013; Pereboom 2009). To explain, briefly: where the ‘sign’ (which is different in different author’s examples)^2^ indicates to Black (or whoever or whatever is the counterfactual intervener) that Jones (or whoever it happens to be) is about to decide to do something contrary to Black’s wishes then Black intervenes, thereby limiting Jones’ decision to one possibility only, which is of course congruent with Black’s wishes. Debate has centred on whether the sign, which is not an indication of a decision, but an indication of what Jones is about to decide, nevertheless amounts to evidence that, on the occasion when the sign was present, an alternate possibility was available to Jones. Riding somewhat roughshod over this debate and allowing that the sign does not indicate an alternate possibility (and therefore a flicker of freedom) – because we do not need it too – the twin world condition requires a comparison between S (or Jones or whoever) in W_1_ (where we allow no alternate possibility to exist) with S in W_2_ (where alternate possibilities incontrovertibly exist). Where S in W_1_ engages in the same action (E) as S in W_2_ then this is sufficient to ascribe moral responsibility to S in either world.^3^In short, we have available to us the means of ascribing moral responsibility even where S could not have done otherwise. We do not need, therefore, to draw on any notion of a pre-decision flicker of freedom.

## Conclusion

In conclusion, I aim to have shown that by positioning the principle of alternate possibilities as a sufficient condition for the ascription of moral responsibility, rather than as necessary, PAP_(s)_ amounts to as useful a maxim as PAP for determining moral accountability. It is able to do this without succumbing to Frankfurt-style counter-examples. Moreover, with the addition of the twin word condition, which I have argued is likewise sufficient for an ascription of moral responsibility, we are able to account for why Jones should be held morally accountable. What I have argued is that there is nothing to be gained, in terms of moral instruction, by insisting on PAP as a necessary condition for moral accountability. What is necessary is that either PAP_(s)_ or the twin world condition is satisfied.

By insisting on either of these conditions for the ascription of moral responsibility, we also preserve some role for alternate possibilities, thereby making it necessary that they feature in our moral decision making in some form or other. The necessity of some role for alternate possibilities does not, however, in and of itself, make necessary the clause that S (or whoever) ‘could have done otherwise’. Because of this, the original formulation of PAP is unnecessary.

## Footnotes

1 In the original Frankfurt paper, Jones is written as Jones4, owing the fact that at previous points in his paper, Frankfurt had discussed other versions of Jones (Jones 1–3).

2 In his original article, Frankfurt suggests that a twitch might be used to indicate that Jones is about to decide to do A (as opposed to no twitch for B). Over the years, variations on this ‘sign’ have appeared, such as flushing bright red (Blumenfeld, 1971) or similarly blushing (Widerker 1995), the monitoring of the protagonist’s brain for an unspecified sign (Fischer and Ravizza 1991, and Zagzebski 2000), the initiation of a specified sequence of neuronal excitation (Stump 1996) or a neurological pattern in the brain (Fischer 2002), the subject’s deliberations and intentions (McKenna 2005), or even the occurrence of moral reasoning of a specified force (Pereboom 2000).

3 The twin world condition also avoids getting bogged down in the debate over whether the relationship between the sign and the decision is indicative of determinism or is indeterminate (Ginet and Palmer 2010; Robinson 2012; Shabo 2011).
